# Using Oscillation to Improve the Insertion Depth and Consistency of Hollow Microneedles for Transdermal Insulin Delivery with Mechanistic Insights

**DOI:** 10.1021/acs.molpharmaceut.4c00942

**Published:** 2024-12-03

**Authors:** Fiona Smith, Anna M. Kotowska, Benjamin Fiedler, Edward Cerny, Karmen Cheung, Catrin S. Rutland, Faz Chowdhury, Joel Segal, Frankie J. Rawson, Maria Marlow

**Affiliations:** † School of Pharmacy, University of Nottingham, Nottingham NG7 2RD, United Kingdom; § Advanced Technology Centre, Oakwood Drive, Nemaura Pharma Limited, Loughborough, Leicestershire LE11 3QF, United Kingdom; ∥ School of Veterinary Medicine and Science, The University of Nottingham, Nottingham NG7 2RD, U.K.; ⊥ Department of Mechanical, Materials and Manufacturing Engineering, Faculty of Engineering, University of Nottingham, Nottingham NG8 1BB, United Kingdom; ‡ School of Pharmacy, University College London, London WC1N 1AX, United Kingdom

**Keywords:** microneedles, insulin, insertion, diabetes mellitus, reproducibility, oscillation

## Abstract

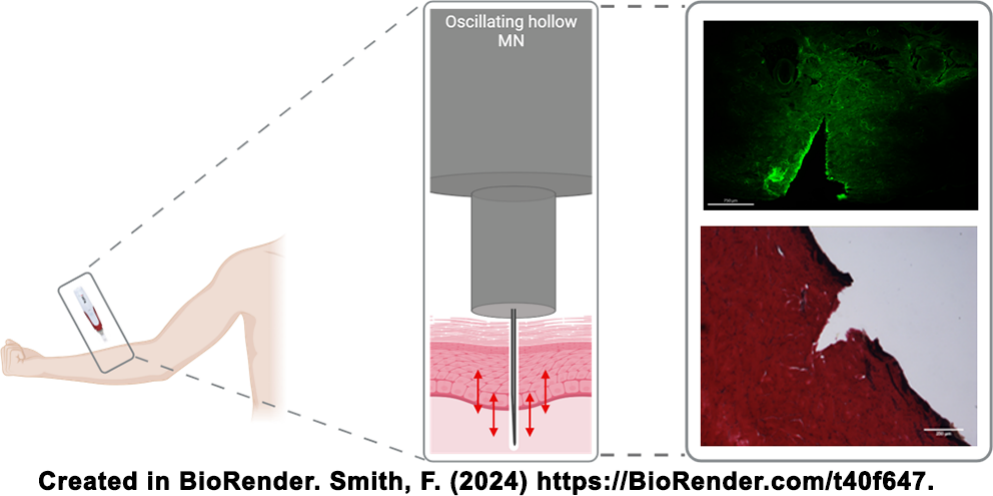

Microneedles (MNs) offer the potential for discrete and painless transdermal drug delivery, yet poor insertion and dosing consistency have hindered their clinical translation. Specifically, hollow MNs are appropriate for the administration of liquid modalities, including insulin, which could prove to be beneficial for patients with type 1 diabetes mellitus. This work aimed to design and manufacture a hollow MN with an improved insertion and delivery profile suitable for insulin administration. *Ex vivo* insertion studies demonstrated that oscillation of MNs upon insertion into skin produced a favorable insertion profile, with reduced variation, compared to static MN insertion. Histological staining showed that this could be due to the repeated motion of the oscillating MN disrupting elastic fibers in the dermis. Additionally, permeation studies demonstrated that increased quantities of insulin were able to permeate the skin when oscillation was employed compared to static MN insertion. This study has shown that oscillation is a valuable tool in improving the transdermal delivery of insulin via a single hollow MN *in vitro*. Moving forward, *in vivo* studies should be completed to gain a fuller understanding of the benefits of the oscillation of MNs on transdermal drug delivery.

## 1 Introduction

Diabetes Mellitus (DM) is a metabolic illness characterized by chronic hyperglycemia associated with impaired insulin secretion or action.^1^ The exact pathophysiology of DM has led to the classification of several subtypes of DM. In type 1 DM (T1DM), previously referred to as juvenile diabetes, the pancreas fails to produce adequate insulin necessary to control glucose homeostasis.^2^ First-line treatment is insulin replacement therapy, which is commenced from diagnosis.^3^ Most commonly, insulin is administered via a regimen of multiple daily subcutaneous injections, consisting of basal and rapid-acting insulin analogues to mimic basal and postprandial insulin release in healthy individuals. Previous research has demonstrated overall poor compliance and adherence to injectable insulin regimens, with pain and the inconvenience of injectable formulations being key contributors.^4−6^ Increasingly, insulin pumps are used by patients with T1DM to reduce the injection burden associated with insulin delivery. However, the use of insulin pumps requires the regular insertion and removal of a cannula into the skin, with a length of 4.5–19 mm, which may be distressing for some patients. As such, there is an unmet clinical need for patient-centric insulin delivery.^7^

Microneedles (MNs), which can be considered as needle-like structures with a height commonly equal to or below 1000 μm, were first considered to offer potential benefits as a medical device for drug delivery in the 1990s.^8,9^ By transiently puncturing the *stratum corneum* of the skin, the transdermal delivery of complex drug molecules that do not favor passive diffusion through the skin is possible.^10,11^ Moreover, owing to the miniature nature of MNs, administration is considered painless and more discrete compared to subcutaneous injections. Since then, MNs made from a vast array of materials using unique manufacturing techniques have facilitated the development of several classes of MNs. Of particular interest in insulin administration are hollow MNs, which feature a bore, allowing the through-flow of liquids.^12^ This will theoretically negate the need for the reformulation of licensed insulin suspensions. Early studies from the Prausnitz group highlighted the feasibility and improved pharmacokinetic profile of insulin administered via hollow MNs, initially in rodents before graduating to human participants.^13,14^ More recently, Luo et al. developed a closed-loop device incorporating hollow MNs with an electroosmotic pump. Results from rodent *in vivo* studies highlighted the reduction in blood glucose fluctuations, but that scale-up in humans may be challenging.^15^ Additionally, Li et al. have exploited advances in additive manufacturing techniques, using static optical projection lithography to 3D-print hollow conical MNs suitable for insulin delivery in mice.^16^ Despite positive initial findings, once again, scale-up from mice to humans will be complex, including the necessary dose and volume increases and potential pharmacokinetic differences.

Despite considerable research into insulin administration using both hollow and other MN subtypes, progress toward a clinically approved device appears to have stalled. While the MicronJet600, a device featuring three hollow MNs, has received FDA approval for the administration of drugs already approved for intradermal delivery, an equivalent outcome has not been achieved for insulin delivery using the MicronJet600, even with two clinical trials to date.^17^ As highlighted in a review article by Smith et al., insertion and dosing consistency remain significant barriers to the clinical translation of MN devices.^18^ Without conclusive evidence that an MN device can reproducibly deliver its payload, it is extremely unlikely that a device will receive approval for use from the regulatory bodies.

An interesting avenue for MNs is their use in the cosmetic sector. Typically, solid MN devices, such as the SkinPen Precision System and MTS Roller, are employed to rejuvenate skin.^19,20^ Of these, multiple devices incorporate mechanisms allowing the needles to oscillate, including the Dermapen.

Oscillation, sometimes referred to as vibration in the literature, combined with MN technology, has been incompletely explored by other researchers in this area. In 2019, Al-Mayahy et al. demonstrated that the use of the Dermapen for pretreatment improved the permeation of imiquimod through porcine skin.^21^ Building on this, in 2020, Sabri et al. revealed that the oscillation of the Dermapen conferred a benefit beyond simply puncturing the skin to improve drug permeation.^22^ The work compared two MN devices, the Dermastamp (no oscillation) and the Dermapen, finding that the Dermapen produced superior insertion efficiency and insertion depth. Other researchers have taken inspiration from the mosquito proboscis, applying vibration to the skin to reduce the force required for reproducible penetration.^23,24^ These works demonstrated the potential benefits of the use of oscillation, laying the foundation for the further work presented here on the mechanistic determination of how oscillating MNs improve transdermal drug delivery.

Initially, in this work, a cosmetic MN device featuring an array of solid MNs that oscillate was thoroughly characterized to elucidate the design characteristics that contribute to the beneficial insertion profile. Later, a single hollow MN capable of oscillating while administering insulin was designed and manufactured to explore whether oscillation aids the consistent and reproducible delivery of transdermal insulin. Data produced here demonstrated that the oscillatory effect of the MNs has a physiological effect on the skin, likely disrupting elastin, thus, improving the MN insertion profile. As a result, increased quantities of insulin were able to permeate the skin, providing affirmation that oscillation may be a valuable tool for improved drug delivery with hollow MNs.

## 2 Experimental Section

### 2.1 Materials

NovoRapid vials (insulin aspart) were purchased from Radford Road Pharmacy, Nottingham. Human recombinant insulin was purchased from Merck Life Sciences Ltd. Fluorescein isothiocyanate (FITC) was purchased from Fluka Biochemika. Dimethyl sulfoxide (DMSO) and acetonitrile (HPLC grade) were purchased from Fischer Scientific. Phosphate buffered saline (PBS) tablets were purchased from Fisher Chemicals. HPLC-grade (ultrapure) water (18.2 MΩ·cm) was available from an SLS Lab Pro PURA-Q+20 Type 1. Teepol solution was purchased from Scientific Laboratory Supplies. Gentian violet 1% w/v was purchased from De La Cruz Products. OCT media was purchased from VWR International Ltd. Methylene blue and fluorescein were bought from Aldrich. Verhoeff’s Elastic Van Gieson (Verhoeff’s EVG) stain kit was purchased from Atom Scientific. The hematoxylin, Weigert’s Iron kit, was procured from ScyTek Laboratories Inc. VeroWhite Plus (RGD835) and Support (SUP705) from SYS Systems were used during 3D printing. Glucose Rx CarePoint 31-gauge, 8 mm insulin pen needles were manufactured by DiME Ltd. and purchased from Chemist4U. Sodium bicarbonate, ethylenediaminetetraacetic acid, and indium tin oxide-coated glass slides were purchased from Sigma-Aldrich. D-Squame standard sampling discs were purchased from Clinical and Derm LLC. Untreated porcine skin was procured from Outwood Farm, Cheadle, and stored at −20 °C before use.

### 2.2 Cosmetic MN Device

The cosmetic MN device used throughout this work resembles the Dermapen device. The device is typically used with disposable arrays of solid conical MNs, which are available with 1,3,7,9,12, or 36 MNs, arranged in lines to form a circular array. MN length can be adjusted between 250 and 2000 μm once attached to the device. The cosmetic device is similar to the Dermapen, which can oscillate the MN arrays at 5 different speeds selected on the device itself (133.3, 166.7, 200, 233.3, 266.7 Hz calculated from 8000, 10000, 12000, 14000, and 16000 rpm).^25^ To characterize the insertion profile for this device, a range of *ex vivo* insertion studies (methodology detailed in Section 2.5) were conducted, including evaluating the differences in insertion depths achieved at different MN lengths and speeds of oscillation. Throughout this work, the oscillation speeds tested were high, medium or low.

### 2.3 Design, Manufacture, and Assembly of Single Hollow MNs

**Figure 1:**
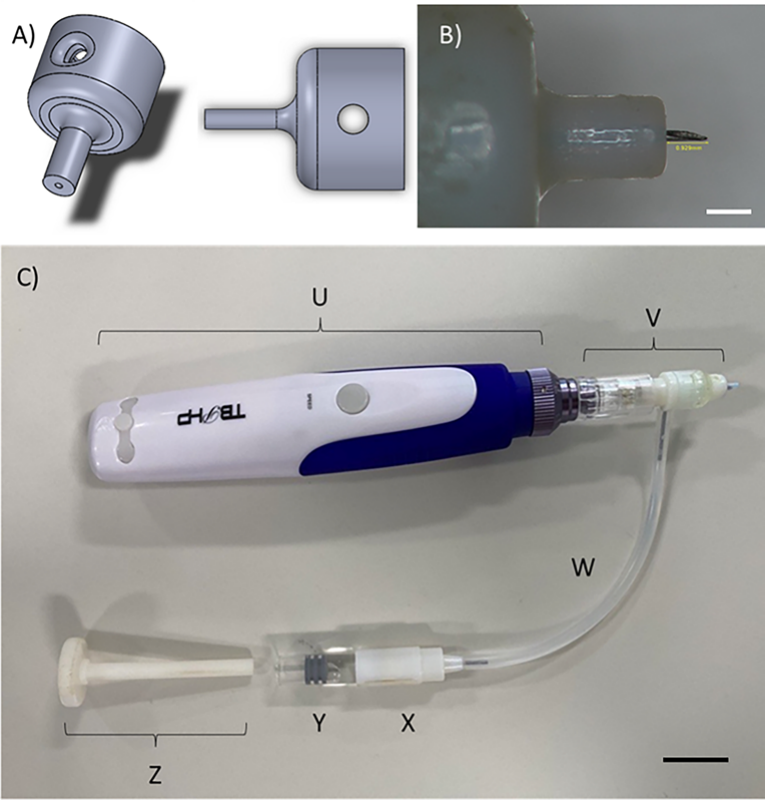
Image A) demonstrates the design of the external casing to be attached to a hypodermic needle, producing a single MN while causing no loss to the needle’s structural integrity. Image B) is an individual hollow MN produced with a hub width of 2 mm and an anticipated needle length of 1000 μm, measuring 929 μm after assembly. Scale bar is equivalent to 1000 μm. Finally, C) shows the cosmetic device modified to include a single hollow MN that can deliver variable volumes of fluid from an external reservoir into skin. Labeled parts include U) the original device casing containing the oscillating mechanism, V) a single hollow MN attachment, W) tubing to connect MN attachment to the reservoir, X) drug reservoir casing, Y) drug reservoir, and Z) plunger. Scale bar is equivalent to 2000 μm.

Initially, an 8 mm, 31-gauge insulin pen needle was drawn to scale in SolidWorks 2019 (Dassault Syetèmes). Thereafter, an external hub was designed that could be attached to the insulin pen needle, making a single hollow MN with an adjustable needle length (Figure 1A). The hub was 3D printed (Objet30 Prime, SYS Systems, UK) using VeroWhite Plus (RGD835) and Support (SUP705) materials. The hub was attached to the insulin pen needle and secured. A base plate was manufactured into which the MNs could be inserted into, ensuring the exposed needle length was equal to the desired length. Needle length was measured after manufacturing and deemed acceptable to be ±10% of the intended length (Figure 1B).

To manufacture a hollow MN capable of oscillating, the cosmetic MN device was remodeled. As shown in Figure 1C, a single hollow MN was joined to an attachment designed to be used with a cosmetic device. This part was attached to the original device body, which contains the mechanism required for the oscillation (labeled U in Figure 1C). An intermediate part, incorporating a screw thread complementary to the insulin pen needle’s screw thread on one end, was manufactured. The remaining exposed end fit snugly over the solid MN array on the device attachment (V). Between the two ends was a dividing wall and, importantly, a hole on the external wall, which a 21-gauge needle could fit through. The needle was secured in place, such that the needle bevel faced down toward the MN attachment. Attached to the other side of the 21-gauge needle was tubing that connected to a drug reservoir (W). At the reservoir, a second 21-gauge needle was inserted into a glass vial, allowing a second port for the attachment of the tubing (X and Y). This setup allowed fluid to flow from the reservoir and through the oscillating hollow MN with the use of a plunger (Z).

To ensure that the volume being injected was known and quantifiable, circular discs that fit into the glass vial were also 3D printed. These were calibrated and labeled so that each disc was equivalent to a known volume when the plunger was fully inserted.

### 2.4 Synthesis of FITC-Insulin

A buffer solution containing 8.4% w/v sodium bicarbonate and 0.058% w/v EDTA in deionized water was made to a pH of 8.5. Lyophilized recombinant human insulin (195 mg, 34.4 μmol, 1 equiv) was reconstituted in 40 mL of the buffer. FITC (26.8 mg, 68.8 μmol, 2 equiv) was dissolved in 4 mL of DMSO before being added dropwise into the gently stirring buffered insulin solution. The molar ratio of FITC/insulin was 2:1. The solution was left to stir for 24 h at room temperature, protected from light.

After 24 h, the FITC-insulin product was purified. First, the solution was concentrated through the use of centrifugal filtration against a molecular weight cutoff of 3000 Da. The remaining solution was passed through a Sephadex PD10 size exclusion chromatography column (PBS eluent) and collected in 0.5 mL fractions. Finally, the samples were dialyzed against PBS for 72 h, with two buffer changes, while being protected from light. FT-IR and HPLC were employed to confirm the conjugation (Figures SI1 and SI4) and CD to confirm that the secondary structure of insulin remained unaffected (Figure SI1). The resulting FITC-insulin was concentrated, lyophilized, and stored in a −20 °C freezer until use.

### 2.5 *Ex Vivo* Insertion Studies

Frozen porcine skin was defrosted at room temperature, and excess hair was removed with microscissors before use. The skin was placed on a cork mat to provide support, simulating muscle tissue. Individual MNs were manually inserted while the skin was held taut using the thumb and forefinger. Unless otherwise stated, MNs inserted into the skin had a needle length of 1000 μm. Insertion of the MNs lasted for 10 s before removal.

To obtain the microchannel depth, a 1% w/v gentian violet solution was immediately added to the skin after the MNs were removed. The dye was left in situ for one h, allowing diffusion through the microchannels, after which excess solution was carefully removed from the surface of the skin.

Thereafter, samples were flash-frozen in liquid nitrogen, mounted with optimal cutting temperature (OCT) compound, and cross-sectioned using a cryostat (Leica CM3050 S Research Cryostat, UK). The microchannels were identified, and channel depth was measured using an optical microscope.

To examine the distribution of a liquid delivered by a single hollow oscillating MN, 100 μL of FITC-insulin was injected into the skin. Samples were flash-frozen and sectioned before being analyzed with an EVOS M5000 imaging system. Both brightfield and green fluorescent protein (GFP) (482 nm excitation, 524 nm emission) channels were used to image the skin and FITC-insulin independently before the images were overlaid.

### 2.6 Assessing the Effect of Biaxial Strain with Oscillation

The biaxial stretch rig developed by Sabri et al. was used to understand the effect of applying varying levels of strain to the skin on the insertion depth achieved by MNs.^22^

Porcine skin was defrosted, and excess hair was removed before being placed into the rig and secured. The porcine skin was subjected to three levels of strain: 1.00, 1.0625, and 1.125, determined by stretching the skin biaxially by 0 mm, 2.5 mm and 5 mm, and measured using callipers.

*Ex vivo* insertion studies using 1% w/v gentian violet solution were conducted while the skin remained in situ, as described in Section 2.5.

### 2.7 Histology Staining (Hematoxylin and Eosin (H&E), Verhoeff’s EVG, and Wiegert’s Van Gieson Stains)

Histological staining was completed on skin sections that underwent treatment with either oscillating solid MNs or an oscillating hollow MN at low, medium, and fast speeds, along with static hollow MN insertion or no insertion. Three repeats were completed for each treatment group. Samples were prepared following the method in Section 2.5, and several different stains were employed. Various histological features in the skin were detected, as shown in Table 1.

**Table 1:** Histological Stains used, Including Tissue and Cellular Details Identified Using Special Stains and their Associated Colour Changes

Stain type	Histological skin features identified
H&E	Nuclei–dark purple
	Cytoplasm/collagen–pink
Verhoeff’s EVG	Collagen–red
	Elastic fibres–black
	Other–yellow
Wiegert’s Van Gieson	Collagen–red
	Nuclei–black
	Other–yellow

Haematoxylin and eosin (H&E) staining was completed using the following procedure. First, slides with skin sections attached were submerged in Harris hematoxylin solution, then washed, dipped in acid-alcohol solution and then washed again. Next, the slides were dipped in Scott’s tap water and then washed again, followed by immersion in 1% w/v eosin solution, and washed. Thereafter, slides were dehydrated via increasing concentrations of ethanol solution (50%, 70%, 90%, and 100% v/v) for 2 s each, followed by an additional 100% ethanol. Slides were then placed in fresh xylene for 2 min before being transferred to finishing xylene. Dibutyl phthalate polystyrene xylene (DPX) was dropped onto a coverslip, which was lowered onto the relevant slide and left to dry.

Verhoeff’s EVG staining was completed using the following procedure. First, solution A was prepared by mixing equal parts of 5% w/v alcoholic hematoxylin solution with 10% w/v ferric chloride solution and adding Lugol’s iodine. Slides were coated in solution A before being rinsed with running tap water. Solution B was prepared by the addition of 10% w/v ferric chloride with distilled water, then applied to the slides, and left until features became distinctive to the eye. The slides were rinsed in running tap water before sodium thiosulfate was added to the slides. The slides were further washed before Van Gieson counterstain was applied. The sections were dehydrated using ethanol, and coverslips were added using DPX.

Wiegert’s Van Gieson staining was completed using the following procedure. First, slides were covered with Weigert’s hematoxylin solution and then washed. Sections were differentiated in an acid-alcohol solution and then washed again before being submerged in Scott’s tap water. Slides were washed before Van Gieson stain was applied, and then the slides were washed again, the sections were dehydrated using ethanol and coverslips were added using DPX.

Skin sections were visualized, and images were captured using a Zeiss Axioplan microscope (Germany), using 2.5× , 5×, or 10× magnification, as applicable.

### 2.8 *Ex Vivo* Insulin Permeation Study

A Franz diffusion cell study was employed to evaluate the permeation of insulin through the skin delivered via the oscillating hollow MN. Full-thickness *ex vivo* porcine skin was defrosted at room temperature, and excess hair and subcutaneous fat were removed and cut into 1 cm × 1 cm pieces. 3 mL of PBS (equal to the volume of the receptor chamber) was added into the receptor compartment before the skin was clamped between the receptor and donor compartments. Franz diffusion cells were placed in a water bath set to a constant temperature (36.5 °C) and stirring speed (840 rpm).

Skin was either injected with 100 or 200 μL of Novorapid insulin via the oscillating hollow MN device (Figure 1C), had insulin pipetted onto the surface of the skin, or was left untreated. When the skin was injected with insulin, the MN was inserted, oscillated for 10 s at either a high speed or low speed, then stopped and partially retracted before the injection of insulin. The MN device was clamped in a 90° position relative to the skin to avoid unintentional movement.

One mL portion of receptor fluid was collected after 15 min, 1, 3, and 24 h and immediately replaced with fresh PBS. Samples were filtered using a 0.22 μm membrane prior to HPLC analysis. After 24 h, the Franz cells were dismantled. The skin surface, donor chamber, and receptor chamber were wiped with sponges soaked in 3% v/v Teepol solution and stored in 5 mL of methanol overnight. Additionally, each skin sample was left to dry for one h before being tape stripped. Fifteen consecutive tape strips were collected and placed in 10 mL of methanol overnight. Both the sponges and tape strips were vortexed for 30 s and filtered using a 0.22 μm membrane, before being diluted 1:10 with PBS, ready for HPLC analysis. Six repeats of each MN treatment were completed.

### 2.9 Quantification of Insulin

HPLC-UV analysis using an Agilent 1100 series instrument (Agilent Technologies, Germany) was completed to quantify insulin postpermeation. A gradient method using a C18 (100 × 2.1 mm) ACE 3 (Hichrom Ltd.) column was employed. The column temperature was set to 35 °C and the detection wavelength of 215 nm (insulin) or 495 nm (FITC) accordingly. Initially, water with 0.1% v/v TFA (A) and acetonitrile 0.1% TFA (B) in a ratio of 80:20 flowed at 0.4 mL/min. 60 μL of the sample was injected, and the mobile phase was adjusted to 55:45 over 10 min, before being held for 2 min, then returning to 80:20 over 3 min, and holding for a further 3 min. Elution time was 8.7 min. Data acquisition and analysis were completed with ChemStation software. Cumulative recovery was calculated using the sum of insulin recovered at each time point compared to the dose administered, taking into account the addition of fresh PBS and the remaining insulin in the Franz diffusion cells.

### 2.10 OrbiSIMS Analysis

Frozen porcine skin was defrosted at room temperature, and excess hair was removed. 100 μL of NovoRapid insulin was injected into the skin using a 1000 μm hollow MN with a high speed of oscillation. Samples were flash-frozen in liquid nitrogen, mounted with an OCT compound, cross-sectioned using a cryostat (Leica CM3050 S Research Cryostat, UK), and placed on indium tin oxide-coated glass slides. Samples were stored in a −20 °C freezer before analysis.

For the acquisition of the OrbiSIMS images, a 20 keV Ar_3000_^+^ analysis beam of 20 μm diameter, was used as the primary ion beam. Ar_3000_^+^ with a duty cycle set to 4.7% and GCIB current was 220 pA. The image was run on an area of 1500 × 2500 μm using random raster mode. The cycle time was set to 200 μs. Argon gas flooding was in operation to aid charge compensation, and pressure in the main chamber was maintained at 9.0 × 10^–7^ bar. Spectra were collected in both positive and negative polarity in the mass range *m*/*z* of 75–1125. The injection time was set to 500 μs. Mass-resolving power was set to 240,000 at *m*/*z* 200. The measurement lasted one scan.

For the acquisition of a liquid metal ion gun (LMIG) ToF-SIMS image, a 30 keV Bi_3_^+^ primary beam was used. LMIG current was 0.05 pA. The ToF image was run on an area of 1500 × 2500 μm using random raster mode. The cycle time was set to 400 μs. The measurement lasted one scan, with 10 frames per scan.

### 2.11 Statistical Analysis

Results were reported as a mean value with standard deviation (SD) or standard error of the mean (SEM) (biological repeats only). Statistical calculations were performed using GraphPad Prism 10 software (IBM, USA). A one-way ANOVA followed by a Tukey posthoc test was applied to compare data groups. A statistically significant difference was denoted by the *p*-value <0.05.

## 3 Results and Discussion

### 3.1 *Ex Vivo* Characterization of Microchannel Production Using Commercially Available Hollow and Solid Cosmetic MN Devices

Initially, the screening of commercially available hollow MN devices using static insertion was carried out. The insertion depths achieved by these commercially available hollow MN devices were poor relative to the total needle length despite the varying needle lengths tested (Figure SI5). These MN devices were not progressed due to the lack of complete insertion of the MN.

**Figure 2:**
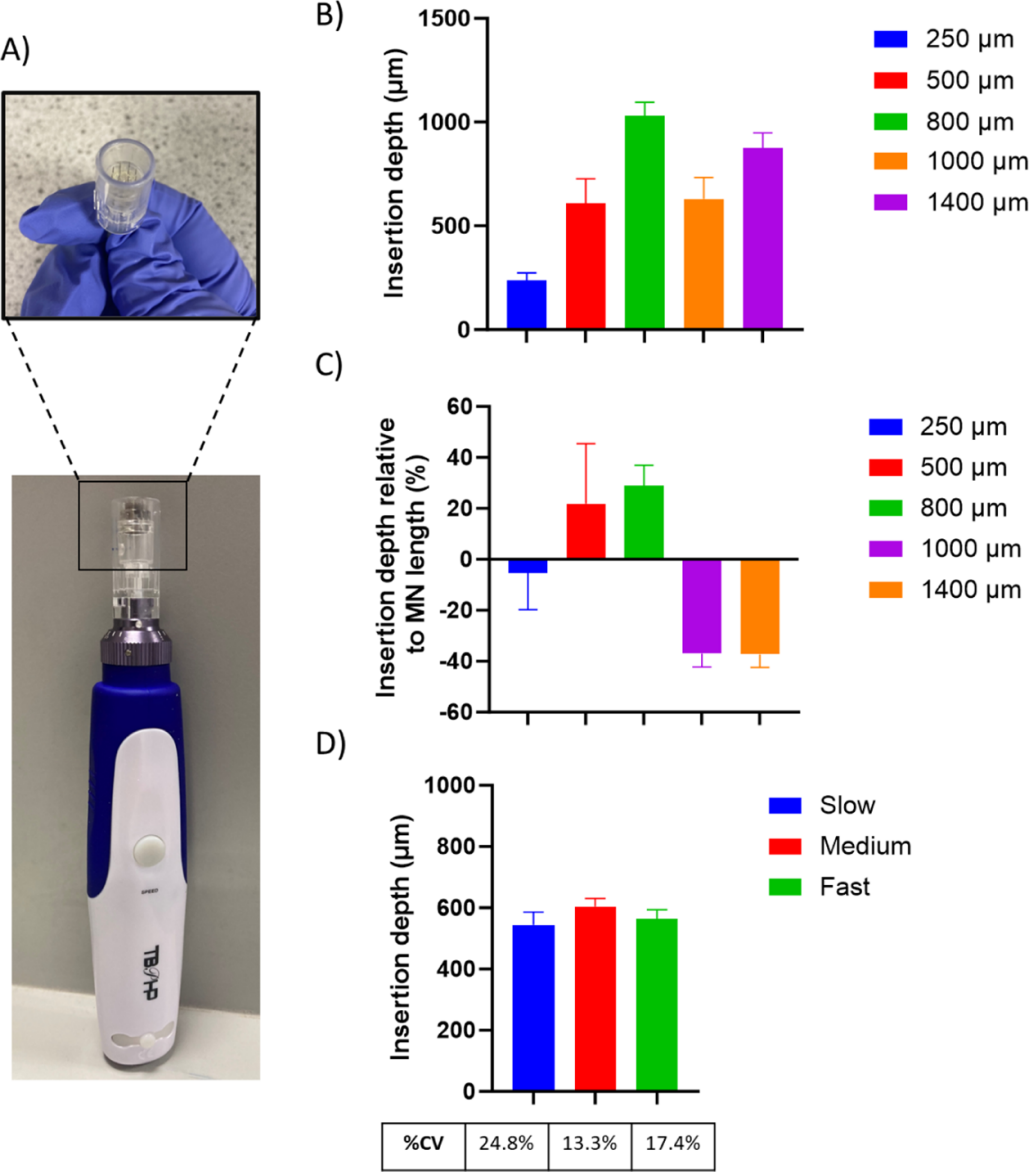
Image A) shows the cosmetic device employed in these studies featuring a removable array of solid MNs. Graph B) shows an *ex vivo* study of the depth of insertion of the solid MNs set to a range of different needle lengths with an array of 12 needles at a fast oscillation speed (mean ± SEM, *n* = 4). Graph C) shows the proportion of the MN that was successfully inserted into skin, as determined poststaining in an *ex vivo* insertion study, normalized to the MN length, using a cosmetic device with an array of solid MNs (mean ± SEM, *n* = 4). Graph D) shows the effect of oscillation speed on the insertion depth achieved by an array of 12 MNs using the cosmetic device in *ex vivo* porcine skin (mean ± SEM, *n* = 10).

As mentioned previously, it was hypothesized that oscillation may be a beneficial accessory to improve the overall MN insertion profile. First, characterization of the microchannels produced using the solid MNs designed for an oscillating cosmetic device was conducted (Figure 2A). The insertion depths achieved by the solid MNs at 5 different MN lengths (250, 500, 800, 1000, and 1400 μm), using the fastest speed of oscillation, are shown in Figure 2B. Unfortunately, static insertion of the MNs at the same lengths was not possible, as the plastic casing surrounding the MNs (Figure 2A) stops the MNs from being exposed while the device is switched off.

It was observed that when the MN length was set at 500 or 800 μm, the insertion depth achieved was deeper than the total MN length, as shown in Figure 2C. Considering previous MN literature where static MNs have been inserted into *ex vivo* skin, this is unusual as a typical insertion depth of around 60% of the total MN length is observed.^26^ In part, this could be due to the outer plastic casing that encompasses the array of MNs that encounters the skin upon insertion. This may apply a degree of strain to the skin, reducing the natural undulations and increasing the insertion depth achieved. A similar design can be observed in multiple applicators that have been designed for use with MN devices, further suggesting the potential benefit.^27^ Moreover, work by Martanto et al. demonstrated that needle retraction can increase flow conductivity in skin tissue, which can be visualized by the spreading of an injected dye.^28^ Effectively, with the oscillating MN device used in this work, the MNs are repeatedly retracted, increasing flow conductivity. Given the methodology employed allows one h for dye permeation, it could be that the oscillation is facilitating excessive dye diffusion, leading to a measurement of insertion depth to be somewhat larger than the total MN length.

Also seen in Figure 2C, the MNs with lengths of 1000 and 1400 μm did not follow this trend. In both instances, the insertion depth achieved by the arrays at these lengths was considerably below the full length of the MN, around 62%. This may be due to incomplete retraction of the MN from the skin before reinsertion. Alternatively, a study by Yang and Zahn previously showed that while oscillation can reduce the insertion force required, a reduction in insertion depth is also observed.^29^ It is thought that this is because the skin is more likely to tear earlier during the insertion process. This is further supported in work completed by Kang et al., who compared the insertion of polymeric microneedles of two different heights using different speeds of vibration.^30^ While insertion depth increased with the speed of vibration, the insertion depth achieved was consistently less than the MN length.

It remains unclear why the insertion depth would suddenly decrease when longer MNs are employed. Regardless, a MN length of 1400 μm may be considered inappropriate, as pain receptors may be stimulated, losing the trademark painlessness associated with MNs.

Despite the incomplete insertion, a MN length of 1000 μm was selected moving forward. In part, a 1000 μm MN was selected after the DOE concluded that needle length to be a significant factor in the achievable insertion depth (see Figure SI6). With a 1000 μm MN, assuming that the skin could be successfully pierced, the MN would insert into the dermis, facilitating the systemic uptake of insulin.

The effect of the speed of oscillation on the insertion depth was also examined. The slowest, medium, and fastest speeds of oscillation (see Section 2.2) were selected and tested using an MN length of 1000 μm. Figure 2D shows that although there was no statistically significant difference in the insertion depths achieved with varying speeds, the variation in insertion depth was reduced when the medium and fast speeds were selected compared to the slowest speed.

While these initial results further confirmed that oscillation may provide a benefit, solid MNs are not a suitable drug delivery platform for liquid drugs with a narrow therapeutic window, such as insulin. To overcome this, a method to manufacture hollow MNs was introduced.

### 3.2 Manufacture of Hollow MNs

The manufacture of hollow MNs remains a challenge in the field owing to the need for specialist equipment, high costs, and the technical skill required for successful production. Additionally, given the nature of research and design work requiring multiple samples for characterization, a different method to produce hollow MNs was employed. By 3D printing an attachment to fit over a hypodermic needle, single hollow MNs were successfully manufactured.

A full factorial design of the experiments further indicated that the area of the MN casing interfacing with the skin surface had no statistically significant effect on the insertion depth achieved (Figure SI6). Additionally, the angle of insertion of the MN into the skin using a 1000 μm MN was explored. Data shown in Figure SI7 demonstrate that insertion depth increased toward perpendicular insertion. As such, a 90° angle of insertion was maintained throughout this work.

### 3.3 *Ex Vivo* Characterization of Microchannel Production Using Oscillating Hollow MNs

The single hollow oscillating MN was tested to understand whether the same characteristics observed previously with the oscillating solid MN arrays were observed here.

**Figure 3:**
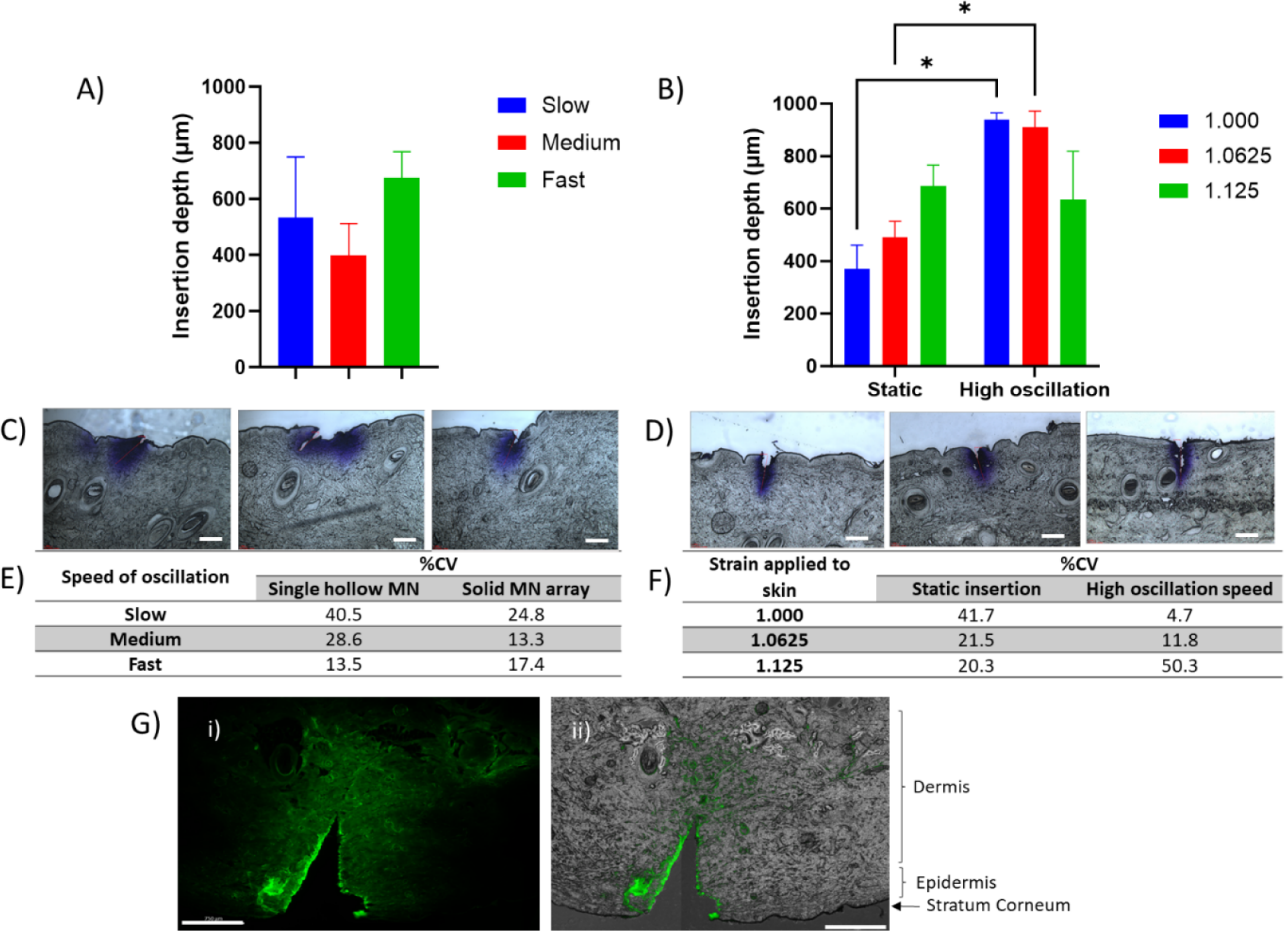
Image A) shows the effect of oscillation speed on the insertion depth achieved by a 1000 μm single hollow MN (mean ± SD, *n* = 3). Figure B) demonstrates the difference in insertion depth achieved with a single hollow MN with a needle height of 1 mm when different levels of strain are applied with and without oscillation of the MN (mean ± SD, *n* = 3). Statistically significant differences were observed between static and oscillating insertion at two levels of strain: 1.000 and 1.0625 (*p* = 0.0105 and *p* = 0.0352, respectively). The skin sections evidenced in C) show the channel created upon insertion of the MN at different speeds, while those in image D) show the channels formed by an oscillating MN at high speed with different levels of strain applied (insertion depths 693, 607, and 820 μm, respectively). Original scale bar is equivalent to 150 μm and white scale bar is equivalent to 300 μm. Table E) further shows the %CV for insertion depth achieved using a single MN oscillating at different speeds. Table F) highlights the %CV for insertion depth achieved using a static MN or an oscillating MN when different levels of strain are applied to the skin. Image G) shows *ex vivo* porcine skin injected with FITC-insulin using a single oscillating hollow MN (needle length = 1000 μm, injection volume = 100 μL) with (i) GFP channel and (ii) GFP image overlaid with brightfield channel showing details of skin. Scale bar is equivalent to 750 μm.

Initially, the insertion depth achieved by the 1000 μm MN at different speeds of oscillation was tested, as shown in Figure 3A. The insertion depth achieved was closest to the length of the MN when the fastest speed of oscillation was employed. Importantly, the variation associated with the insertion depth was considerably reduced when the fastest oscillation was used compared with the slowest oscillation in combination with the single hollow MN. This trend is much more defined than when the single hollow MN is used compared with the solid MN array.

Previous research has shown that the application of strain to the skin during MN insertion may also be beneficial in improving insertion depth.^31^ Indeed, applicator devices for MNs frequently include a built-in mechanism that will apply strain, and subsequent stretching, to the skin.^27^ As such, a combinatory approach of oscillation and the application of strain was employed to attempt to increase the insertion depth to 100% of the MN length.

We compared the insertion of both a static MN and an MN oscillating at high speed when three different levels of strain were applied to the skin. The oscillation had a statistically significant benefit in terms of the insertion depth compared to that of the static MN at low and medium levels of strain. Interestingly, at the highest level of strain, 1.125, on average the insertion depth appears to be reduced with the oscillating MN (Figure 3B).

Despite this, the most improved insertion depth was observed when no additional strain was applied to the skin (1.000), beyond that of being clamped in the rig, with the use of the 1000 μm oscillating single hollow MN. Given the use of *ex vivo* porcine skin here, it is not unreasonable to assume that a degree of strain would be applied to the skin in living subjects owing to the human skeleton, suggesting that no further application of strain would be required.

The table shown in Figure 3F compares the coefficients of variation (%CV) obtained during both static and oscillating insertions at various levels of strain. Further supporting the improved insertion depth obtained when no additional strain was applied to the skin, the coefficient of variation was reduced to 4.7%, the lowest %CV achieved. As strain increased, variation also increased when oscillation was used, likely related to the loss of consistent insertion despite the MN length staying constant.

Figure 3D shows images of the corresponding skin sections from the skin that have been treated with the oscillating MN under strain. Compared with the images in Figure 3C, in which the biaxial rig was not used, the microchannels appear well-defined and perpendicular to the surface of the skin. Although not shown here, it was observed that when the oscillating MN was applied to the skin at the highest level of strain, at times there was considerable damage to the skin.

To assess whether the improved insertion depth may also confer successful drug delivery, FITC-insulin was successfully synthesized and characterized, as shown in Figures SI1 and SI4. FITC-insulin was injected into the skin using an oscillating hollow MN. Figure 3G shows a clear insertion point produced by the MN. When examined under a GFP channel (Ex: 482, Em: 524), FITC-insulin could be observed around the microchannel, confirming the successful intradermal delivery of insulin using the adapted device.

### 3.4 Mechanistic Effect of Oscillation on MN Insertion in *Ex Vivo* Skin

Histological stains were employed to understand whether the oscillation of the MN device alters the skin tissue in a way that differs from damage caused by static MN insertion. Verhoeff’s EVG and Wiegert’s Van Gieson were used alongside H&E staining to give an insight into any alterations in elastin and collagen fibers that are responsible for the viscoelastic properties of the skin. Verhoeff’s EVG stain is particularly useful for understanding the cause of disruption to the skin, given the synergistic relationship between collagen and elastin in the skin.^32^ Collagen and elastin both provide mechanical support to the skin. In the first instance, when skin is subjected to strain, elastin will provide support; however, as the strain increases, collagen will unfold, providing stiffness to the skin.

**Figure 4:**
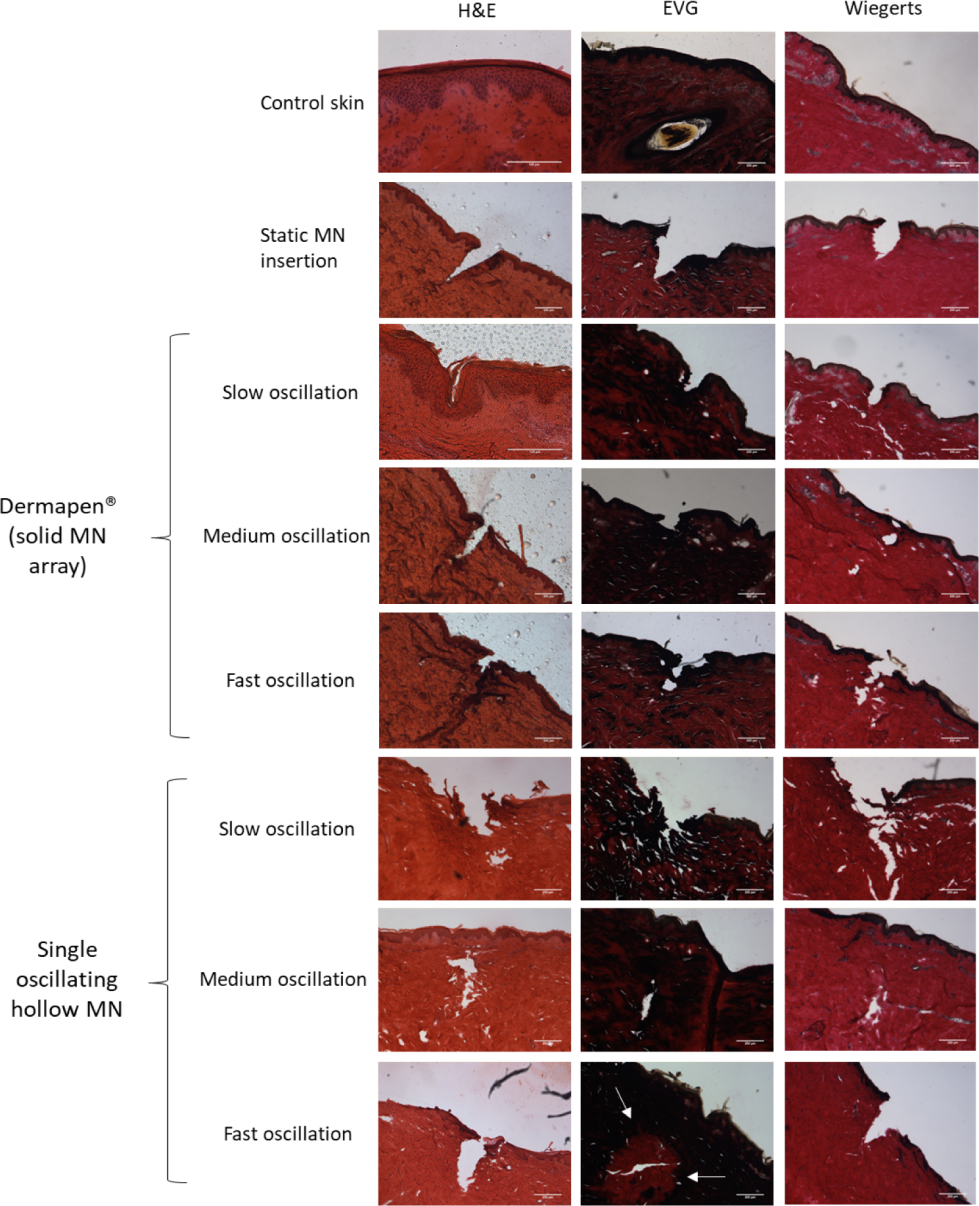
A) Porcine skin cross-sections prepared with H&E, EVG, and Wiegert’s histological stains, to visualize anatomical features, including collagen and elastin. Skin has either been treated with the cosmetic device with an array of solid MNs at varying speeds of oscillation, a single oscillating hollow MN with varying speeds of oscillation, a static MN, or no treatment (control skin).

The use of a static MN reiterates that insertion into the skin is possible, as shown in Figure 4, which is identifiable from the clear break between adjacent cells. Insertion ends in the dermal layer of skin, as anticipated based on the MN length. Here, the viscoelasticity conferred from the elastin and collagen has been overcome by the MN; however, the distribution of both fibers appears to remain constant throughout the sample.

The cosmetic device with an array of 12 solid MNs was tested to understand the effect of different speeds of oscillation on the skin. First, the slowest speed of oscillation was used. Interestingly, considering the H&E-stained sections, it was unclear whether the skin had been punctured by the MN or compressed to fit around the MN, as the *stratum spinosum* and *stratum basale* layers did not appear to be disrupted. However, the *stratum corneum* appears to have been disrupted, which would facilitate the movement of the drug through the skin. Conversely, the EVG and Wiegert’s stained sections suggested the skin had been successfully punctured,;however, the insertions did not seem as well-defined as with the static MN insertion.

Next, medium-speed oscillation was tested. A more defined insertion was present, with insertion further into the skin compared to slow oscillation. Moreover, it appeared that the MN punctured the dermis. This implies that enough force was provided by the medium speed of oscillation to disrupt collagen and elastin in the skin.

The fastest speed of oscillation showed findings similar to those of the medium speed of oscillation, with insertion into the dermis and a well-defined break in the *stratum lucidium*. There appeared to be more collagen around the entry point of the insertion, which has likely come from the surface of the skin as the MN has been inserted.

Thereafter, the single hollow MN was tested at the same 3 speeds of oscillation to understand whether there is a difference in the insertion between an array of solid MNs compared to a single hollow MN.

Skin sections in which slow oscillation was employed showed that MNs could insert into the skin. However, similar to the observations of slow oscillation with solid MNs, the channels did not seem to be as well-defined as the control samples with no oscillation. Indeed, what appears to be debris from the surrounding skin was present around the opening of the microchannel. Once again, this supports the hypothesis that the speed of oscillation is of key importance in ensuring reproducible MN insertion.

Figure 4 also shows skin sections when fast oscillation with a single hollow MN was used. These images show a clear channel, penetrating the dermal layer of the skin. Of note, the skin sections stained using EVG appeared to show a change in the distribution of elastic fibers around the apex of the channel (highlighted by white arrows). Given that the only difference in the MN treatment was the speed of oscillation, this may suggest that at high speeds of oscillation, elastin within the skin is disrupted. This would cause a loss of the typically high viscoelasticity of the skin, improving the insertion profile. In comparison, there appeared to be no change to the distribution of collagen fibers, which have a higher Young’s modulus compared to elastin.^32,33^

These changes were not identified when sections of skin treated with the fastest speed of oscillation were examined by using an array of solid MNs attached to the cosmetic device. An explanation for this may be in the use of an array, rather than a single MN. As such, the force would have been distributed across all 12 MNs in the array rather than just one MN. Subsequently, the stress applied to the skin by each MN would have been reduced compared to the single MN. The increased stress applied to the skin through one point is likely to have caused more significant disruption to the elastic fibers in the local region.

From Figure 4, it is evident that a degree of damage was inflicted on the skin during the successful MN insertion.

Of concern when disrupting the skin barrier is the risk of infection. Multiple studies have been conducted previously that demonstrated channels produced by MN insertion have rapidly closed after removal of the device, given the inherent viscoelasticity of the skin.^34−36^ Given the hollow nature of the MNs designed and tested here, there should be further investigation into the damage to the skin caused by the hollow MN, including the possible loss of tissue integrity caused by the formation of a skin plug when the MN inserts. While the point of insertion and the channel formed remain relatively small considering the total surface area of the skin, an assessment of whether the oscillation damages the skin less transiently than a static insertion will have to be explored. This was not possible in the study completed here owing to the *ex vivo* nature of the skin. Moving forward, *in vivo* studies on the safety of oscillating MNs and the healing of the skin would be required to satisfy regulatory bodies.

Moreover, the safety of the material used must be considered. Here, the MNs are intended to be inserted into the skin for a short period of time before being removed. They are made from the stainless-steel sheath of insulin needles, therefore producing little risk of an adverse reaction; however, inflammation caused by localized damage of the skin should be further explored *in vivo* before considering clinical translation.

Additionally, although unrelated to safety, not explored here is the perception of pain from insertion and administration of the drug via the MNs. This would be required to support the clinical need for such a device. Previously, Gill et al. and Gupta et al. demonstrated that MNs with a length of up to 1000 μm were less painful upon insertion into the skin than a hypodermic intradermal insertion.^37,38^ Interestingly, Gill et al. demonstrated that even at reduced microneedle lengths of 480 and 700 μm, only 30% of participants reported microneedle insertion as painless. A later study by Li et al. further suggested that MN arrays consisting of MNs with lengths below and even above 1000 μm were acceptable to volunteers in terms of pain, which has been supported by work by Lee et al., who demonstrated a significant improvement in VAS scores with long (4 mm) MNs.^39,40^ More recently, Jeong et al. have collated VAS scores from several studies and concluded there are no significant differences in the VAS scoring with MNs between 180 and 1000 μm.^41^ However, the effect of the oscillation was not explored in these cases; therefore, in the future, a study with human volunteers would be beneficial.

Overall, histological staining provided evidence to suggest that oscillation may be beneficial in aiding MN insertion into skin, likely due to disruption of elastic fibers found within the skin. Additionally, it appears that the speed of oscillation may be a critical factor in optimizing the insertion profile of the MN. This hypothesis is supported by previous work undertaken by Van der Maaden that explored the use of applicators to control the insertion velocity. The work demonstrated that the utilization of an impact-insertion applicator improved the insertion efficiency and reproducibility compared to manual insertion.^42^ In the future, biochemical assays, such as ELISA, could be employed to quantify changes in collagen and elastin with different speeds of oscillation, further supporting the findings presented here.

### 3.5 *Ex Vivo* Drug Delivery and Permeation Study

A permeation study was conducted to quantitatively assess the benefits of oscillation for transdermal insulin delivery across the skin. Following from the findings in Section 3.3, initially a single hollow MN was inserted using the highest speed of oscillation. Given that the application of strain to the skin caused increased variability, the skin was injected while freestanding on a cork mat during insulin administration. The insulin suspension was administered manually via a syringe and plunger, as shown in Figure 2c. Pressure was applied by the thumb of the user during all experiments, aiming for slow and consistent delivery of the insulin. Although outside the scope of this work, further development, initially using a syringe pump, followed by automation of the device to control the flow rate, may further ensure consistency, with the use of force sensors to verify this.

**Figure 5:**
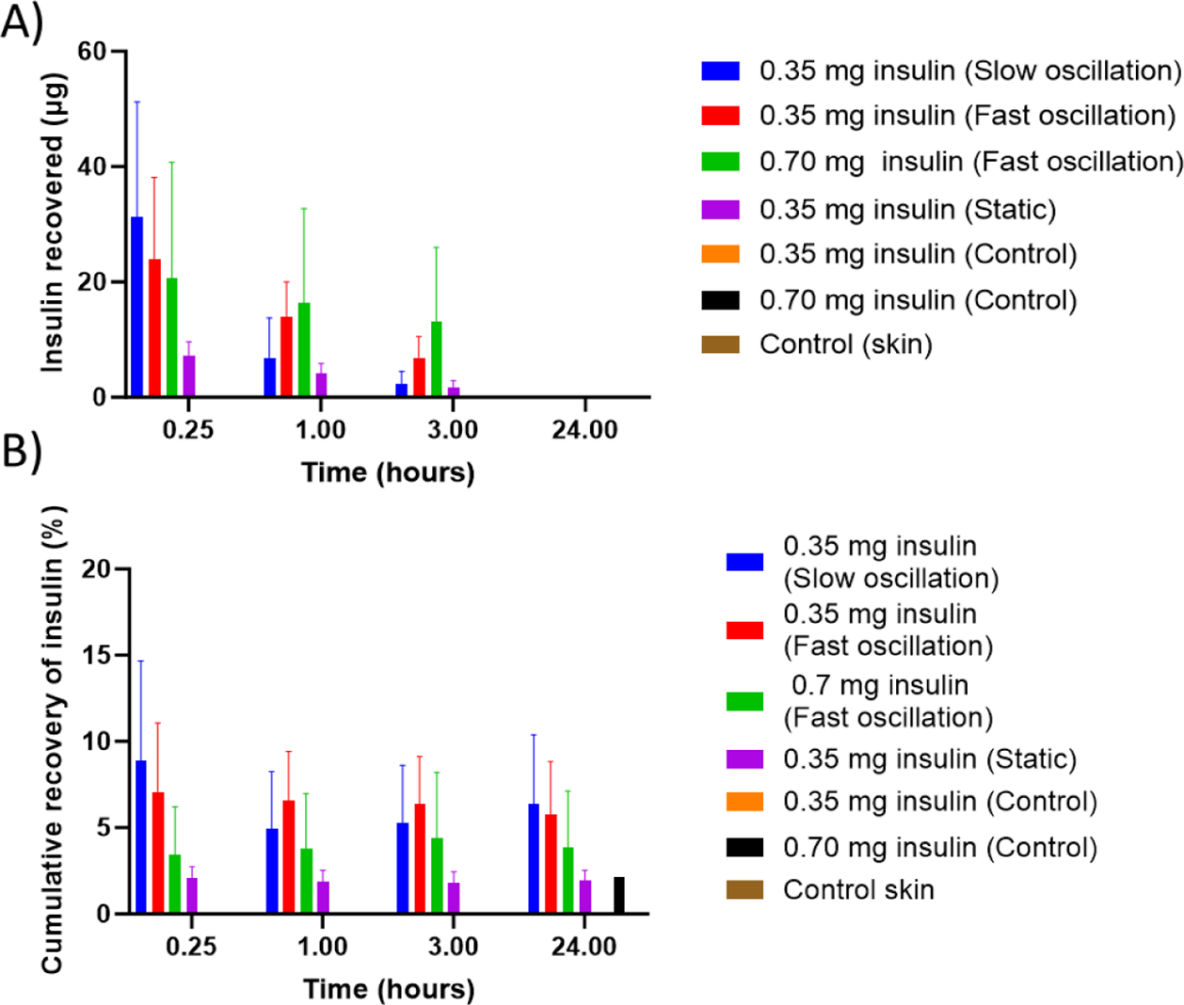
A) Insulin recovered (μg) from receptor fluid in all Franz diffusion cells post injection of 350 or 700 μg NovoRapid insulin using a single hollow MN oscillating at a fast speed, slow speed, and static and controls (needle length = 1000 μm) at 15 min, 1, 3, and 24 h. Initially, slow oscillation produces the highest recovery, yet by 3 h, delivery of 0.7 mg of insulin using fast oscillation is providing the highest recovery. By 24 h, the insulin recovery has dropped to 0, which may be caused by the insulin diffusing back into the skin. Insulin quantified using HPLC. Data expressed as treatment *n* = 6 ± SEM. B) Overview of the cumulative insulin recovery (% theoretical loading) from all Franz diffusion cells receptor fluid post injection of 350 μg or 700 μg NovoRapid insulin using a single hollow MN oscillating at a fast speed, slow speed, and static and controls (needle length = 1000 μm) at 15 min, 1, 3, and 24 h. At 24 h, total recovery was calculated by combining insulin recovered from the receptor fluid, tape strips, and Teepol-soaked sponges. Cumulative recovery remains less than 10% of the theoretical total in all treatment groups and shows an increase when oscillation is used. Insulin quantified using HPLC. Data expressed as *n* = 6 ± SEM.

Both 100 μL (350 μg) and 200 μL (700 μg) of NovoRapid insulin were administered to assess the ability to deliver variable doses of insulin with oscillation. Figure 5 shows the concentration of insulin in the receptor fluid and the cumulative recovery at each time point.

Increased concentrations of insulin in the receptor fluid were observed after 15 min with both treatments. This could be due to the high concentration postadministration, driving diffusion of insulin through the skin into the receptor chamber in a short time frame. Using a fast speed of oscillation, when 350 μg of insulin was dosed through the MN, it was possible to recover 7.07% of insulin after 15 min, compared to 3.42% when the volume was increased. The concentration then reduced over time until, by the 24 h time point, the insulin was no longer detectable. Despite administering twice the volume of insulin, the concentration recovered from the receptor fluid remained similar at most time points. One possible reason for this is that 200 μL is a large volume for intradermal injection. Subsequently, 100 μL of insulin was administered via the MN going forward.

To further understand whether the speed of oscillation is an important parameter, slow oscillation and no oscillation were also tested.

The highest insulin recovery was observed with slow oscillation at 31.20 μg after 15 min. The insulin delivered with no oscillation was over four times lower at the same time point, measuring 7.19 μg. Fast oscillation appears to give a superior profile for the remainder of the study, with higher concentrations at the 1 and 3 h time points. After 24 h, the slow oscillation demonstrated the highest cumulative recovery; however, there was no statistical difference between slow and fast oscillation. The cumulative recovery of the insulin administered with no oscillation is lower in comparison, reaching only 1.91%, suggesting that there is some benefit in using MNs with oscillation.

Comparatively, a study conducted by Xenikakis et al. evaluating insulin delivery with two different hollow MN geometries demonstrated a cumulative recovery of insulin of 4.3% and 6.0%, respectively, after 24 h.^43^ Xenikakis et al. attributed low insulin recovery to the use of full-thickness porcine skin in their work, similar to the study conducted here. Furthermore, when Cheung et al. completed an *in vitro* study evaluating the effect force has on insulin permeation from AdminPatch devices, the cumulative recovery of insulin was in the range of 0.2% to 37.1%, depending on the conditions used.^44^ These studies suggest that the cumulative insulin recovery is consistent with findings from other researchers and that there may be a benefit when exploiting oscillation with an optimized MN geometry.

Further accounting for the incomplete cumulative recovery, it is understood that insulin is most soluble at an acidic pH rather than at physiological pH.^45,46^ PBS was used in the receptor chambers, which have a pH of 7.4 to mimic physiological conditions. It could be argued that it is more likely that insulin remains in the skin or partitions back into the skin later in the study. This could explain the apparent reduction in insulin concentrations in the study.

A further explanation for such a low recovery could be the instability of insulin. The insulin used here is a licensed medicine, NovoRapid. Both the manufacturers and other researchers advise that NovoRapid may be used for up to 24 h after dilution with 0.9% sodium chloride, 5% dextrose, or 10% dextrose at a concentration of 0.05 unit/mL to 1.0 unit/mL when kept at room temperature.^47,48^ Assuming the complete permeation of insulin through the skin in this experiment, the final concentration of insulin would have been considerably higher than this, which could favor aggregation. Moreover, the experimental setup utilized PBS as opposed to other diluents tested by the manufacturer; however, 24 h does not seem an extreme time frame given this advice.

Another consideration is whether the insulin adsorbed onto the glass of the Franz diffusion cells, contributing to the incomplete recovery of the dosing insulin. In the past, it has been well documented that insulin has aggregated on hydrophobic surfaces, such as glass and plastics.^49−51^ This has frequently been explored in a clinical context in relation to whether dose adjustment is required.^52^ In future work, it may be possible to further explore the interaction between insulin and the Franz diffusion cells to understand whether this contributed to the total cumulative recoveries observed.

After the 24 h time point, sponges soaked in Teepol solution and tape strips were used to collect any excess insulin from the surface of the skin. Insulin was not detectable from either the sponges or the tape strips collected from the MN-treated skin. This was not unexpected, as insulin should be absent from the outermost layers of the skin. As the MN length is 1000 μm, the MN should be inserted into the dermis layer of the skin, where insulin is then administered. Moreover, tape strips remove single layers of the *stratum corneum*, with 15 tape strips equating to a depth of around 6 μm. Assuming the successful intradermal delivery of insulin, insulin should not be detected from the tape strip samples.

Figure 5 illustrates the standard error of the mean (SEM) calculated for each time point. These figures show that the SEM was higher when oscillation was used compared with a static insertion, suggesting there is further room for improvement with dosing consistency. During this work, insulin was manually injected after 10 s of MN oscillation. A more automated device may reduce SEM by accurately controlling the duration of oscillation and the rate of infusion, thereby reducing human error. Variation can also be attributed to the use of a Franz diffusion cell setup, including biological tissues. The relationship between the speed of oscillation and insulin recovery is less clear and warrants further investigation.

Previously, it was discussed that hollow MNs may become blocked with a skin plug after a single insertion into the skin, which would prevent the administration of the drug. During this study, MNs were not checked for blockage as, following current clinical guidance, the intention would be that the MNs are for single use only.^53,54^ It was not believed that blockage of the MN was a significant issue in this work, given the successful detection of insulin, the receptor fluid of the FDC.

Overall, these data suggest that there may be a benefit in pairing oscillation with the use of MNs for the delivery of insulin, increasing the deliverable dose. In the future, to further validate the delivery of insulin and explore the distribution of insulin within the FDC, it may be possible to extract the insulin from the porcine skin after the final time point and quantify the insulin.^55^ As an alternative, OrbiSIMS analysis was used to visualize insulin delivery postadministration.

### 3.6 OrbiSIMS Analysis

Secondary ion mass spectrometry (SIMS) has been reported in the literature as a tool for monitoring the delivery of unlabeled active pharmaceutical compounds into the skin from liquid and gel formulations, as well as from MN formulations.^56−58^ Historically, such analysis was limited to imaging relatively small molecules due to intense molecule fragmentation during the data acquisition process. The development of the OrbiSIMS has enabled a more detailed analysis of biological samples, including characterization of proteins at surfaces and profiling the delivery of peptide compounds into the skin.^59−61^

Here, we utilized the capability of the OrbiSIMS to image the delivery of insulin to skin without modifying or tagging the protein.

Sections of full-thickness porcine skin that had been injected with insulin using a single oscillating hollow MN were analyzed to confirm the successful administration and distribution of insulin.

**Figure 6:**
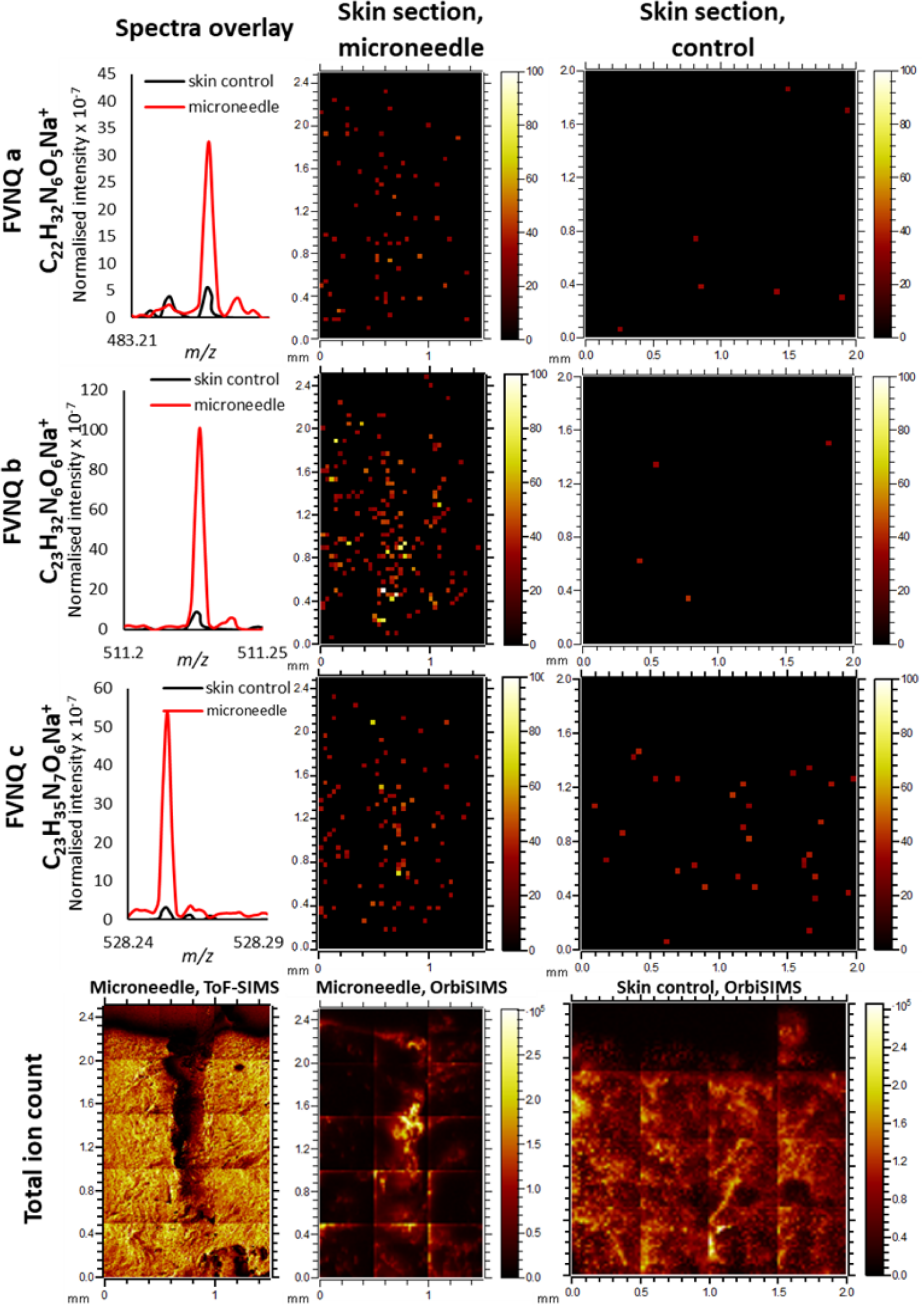
OrbiSIMS imaging of insulin fragments in the skin sections treated with insulin-loaded MNs and untreated skin. Fragments of the insulin sequence, FVNQ, are detected in the OrbiSIMS spectra and are present only at noise levels in the untreated skin control. Both ToF-SIMS and OrbiSIMS images of the MN area are presented to illustrate MN insertion with higher lateral resolution.

Table 2 shows the fragments that were identified during the OrbiSIMS analysis, based on findings from previous work conducted by Kotowska et al., indicating the presence of insulin.^61^ To further confirm these findings, peaks with the corresponding *m*/*z* ratio were also searched in the control skin. Figure 6 shows the spectra of the ions listed in Table 2 in both the control and the skin used for insulin administration. The complete amino acid sequences of insulin are absent (below the noise level) in the skin.

**Table 2:** Peak List of Insulin Fragments Identified in the Positive Polarity Spectrum with their Affiliated Chemical Assignment, *m/z* Value, and Deviation (to Assess the Accuracy of the Assignment)

Description (amino acid sequence)	Assignment	*m*/*z*	Deviation (ppm)
FVNQ	C_22_H_32_N_6_O_5_Na^+^	483.2320	–1.3
FVNQ	C_23_H_32_N_6_O6Na^+^	511.2271	–0.8
FVNQ	C_23_H_35_N7O_6_Na^+^	528.2536	–1.0

The total ion count for ToF-SIMS, presented in Figure 6, clearly shows that the microchannel successfully formed upon insertion of the MN into the skin.

Moreover, the images in Figure 6 illustrate that the fragments associated with insulin have diffused away from the microchannel. In particular, ion b of the FVNQ sequence showed distribution in the skin surrounding the point of administration. This is promising when considering the requirement of insulin to diffuse through the skin and pass into the systemic circulation for the successful control of blood glucose concentrations.

These findings, paired with the permeation study and early characterization work, provide strong evidence for the successful delivery of insulin to the skin.

## 4 Conclusions

Oscillation was identified as a promising feature for improving insertion and dosing consistency for the transdermal delivery of insulin via MNs through a series of *in vitro* assessments. A single hollow oscillating MN of 1000 μm was manufactured by using commercially available products and 3D- printed custom parts. Importantly, the device featured a reservoir, facilitating the adjustment of the injection volume.

The completion of an *ex vivo* insertion study showed that using the fast speed of oscillation increased the achievable insertion depth, while the coefficient of variation reduced. A dual approach combining oscillation with strain was attempted; however, inflicted unacceptable damage to the skin.

To further understand the effect of oscillation on skin tissue, histological staining was completed. Weigert’s, EVG, and H&E stains were selected to understand whether elastin and collagen were altered during *ex vivo* MN insertion. When skin sections were analyzed, it appeared that there was a loss of elastic fibers around the microchannel when a fast speed of oscillation was employed. It is hypothesized that this may be related to the oscillation increasing stress on the skin, damaging the elastin.

Moreover, through qualitative and quantitative pharmaceutical analysis, it was confirmed that insulin was successfully delivered into the dermis of the skin and was able to permeate through the deeper layers of tissue. Indeed, HPLC analysis confirmed that using oscillation facilitated the delivery of 22.3 μg of insulin compared to 6.7 μg with a static insertion of a hollow MN after 24 h. OrbiSIMS analysis gave further insight into the distribution of insulin in the skin immediately postadministration.

Data presented here demonstrate the potential benefit of combining oscillation into an MN device suitable for use by those living with a diagnosis of T1DM. Additionally, this work provides some explanation for the differences observed between static and oscillating MN insertion using *in vitro* models. The next step would be to further validate these findings using an appropriate *in vivo* model as well as collecting additional information on skin healing and pain perception with the oscillation. Overall, considering the growing acceptance of oscillating solid MN devices in the cosmetic industry, it is plausible that an oscillating hollow MN device, with an acceptable safety profile, in the pharmaceutical sector would gain patient acceptance.

## Supporting Information Available

Circular dichroism (CD), FT-IR, and CD of native and FITC-insulin, HPLC method development, HPLC of FITC-insulin, *ex vivo* insertion with commercially available hollow MNs, full factorial design of experiments, and the effect of angle on insertion depth (PDF)
