# Supplementary material for: Using Oscillation to Improve the Insertion Depth and Consistency of Hollow Microneedles for Transdermal Insulin Delivery with Mechanistic Insights
Source: Mol Pharm. 2024 Dec 3;22(1):316–29. doi: 10.1021/acs.molpharmaceut.4c00942 (PMC11707737; doi:10.1021/acs.molpharmaceut.4c00942)
Supplement: Supplementary file 1 — mp4c00942_si_001.pdf [file mp4c00942_si_001.pdf]

## Supporting information

# Using oscillation to improve the insertion depth and consistency of hollow microneedles for transdermal insulin delivery with mechanistic insights

Fiona Smith [a], Anna M. Kotowska [a], Benjamin Fiedler [a†], Edward Cerny [b], Karmen Cheung [b], Catrin S. Rutland [c], Faz Chowdhury[b], Joel Segal[d], Frankie J. Rawson[a] & Maria Marlow[a]*

[a] School of Pharmacy, University of Nottingham, Nottingham NG7 2RD, United Kingdom

[b] Nemaura Pharma Limited, Advanced Technology Centre, Oakwood Drive, Loughborough, Leicestershire LE11 3QF, United Kingdom

[c] School of Veterinary Medicine and Science, The University of Nottingham, NG7 2RD, UK

[d] Department of Mechanical, Materials and Manufacturing Engineering, Faculty of Engineering, University of Nottingham, Nottingham NG8 1BB, United Kingdom

[†] School of Pharmacy, University College London, London, WC1N 1AX, United Kingdom

*Corresponding author: Maria.Marlow@nottingham.ac.uk, Tel.: 0115 8467045

### Circular dichroism (CD)

Before use, the CD (Chirascan VX, Applied Photophysics Limited, UK) was purged with nitrogen gas to deplete oxygen in the system. Reconstituted insulin (both naked and tagged) was diluted 1:200 with HPLC grade water and loaded into a 1 mm high precision quartz cuvette (Hellma Analytics). The temperature was monitored throughout each run and stayed constant between 21.7-21.9 °C. Spectra were recorded between 190 and 280 nm. Step size was 1 nm, bandwidth was 1 nm, count rate was 20,000 and acquisition time was 0.5 seconds. Three repeat scans were completed for each sample, the average calculated and plotted using OriginLab (Pro) 2023. Between runs, the cell was thoroughly washed and dried to avoid contamination and stored in Hellmanex solution after use.

31 FT-IR and CD of native and FITC-insulin

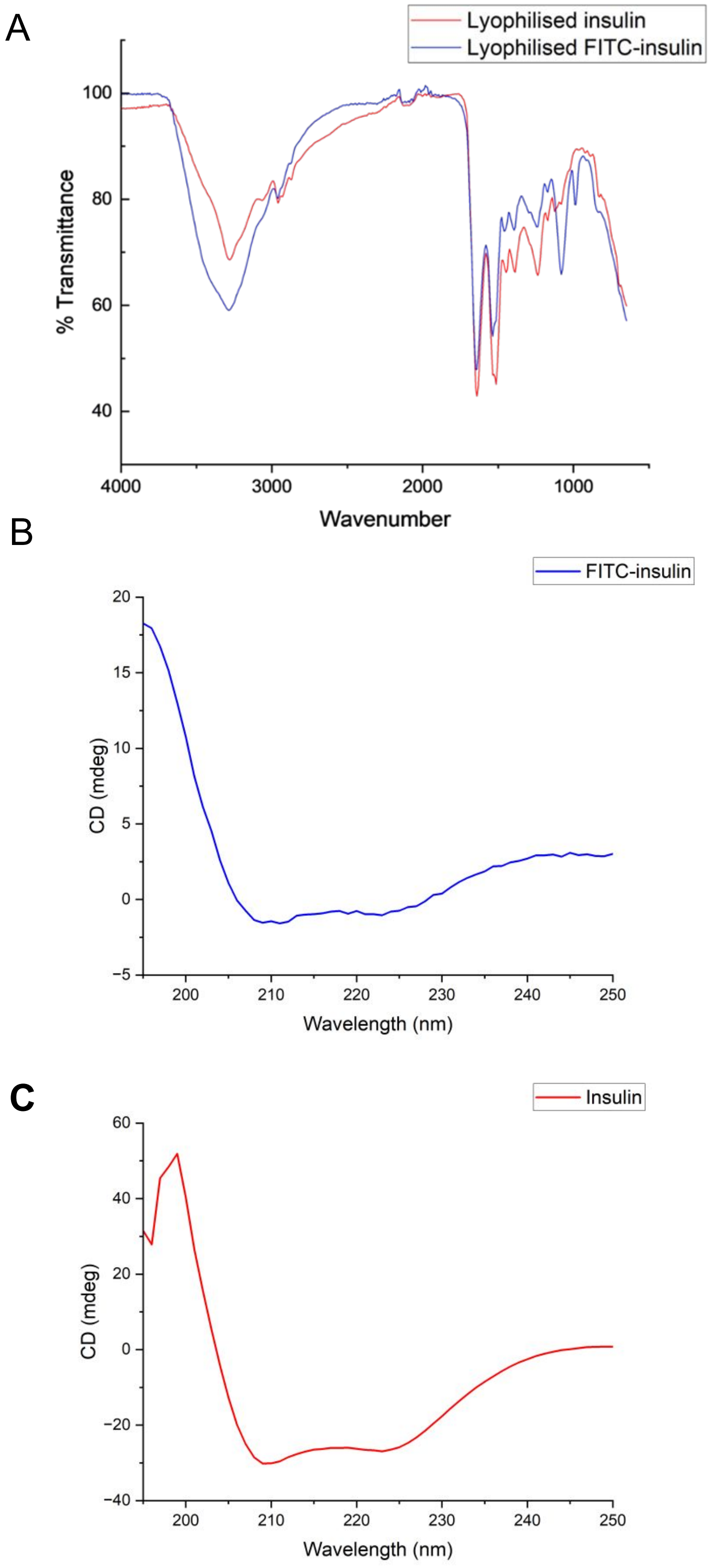

***Figure SI1:*** *Analysis of synthesised FITC-insulin showing A) the FT-IR spectra of lyophilised insulin with FITC-insulin overlaid. Amide peaks are identifiable between 1500-1600 cm$^{-1}$ and 1600-1700 cm$^{-1}$, whilst a peak representing the isothiocyanate groups is present in the FITC-insulin sample at 1080 cm$^{-1}$ and B) CD spectra with peaks identifiable at 208 nm and 222 nm, demonstrating the method used for the synthesis of the FITC-insulin does not detrimentally alter the native insulin structure, shown in C). These analyses confirm the successful conjugation between FITC and insulin without disrupting the insulin secondary structure.*

<u>HPLC method development</u>

Standard curves with concentrations of 0, 1, 3.125. 6.25, 12.5, 25, 50, 100 and 250 µg/mL of insulin, diluted in PBS, were prepared. To assess intra-variability, five standard curves were run on the same day (Figure SI2). Separately, five standard curves of the same concentrations, were run on different days to assess inter-variability (Figure SI3).

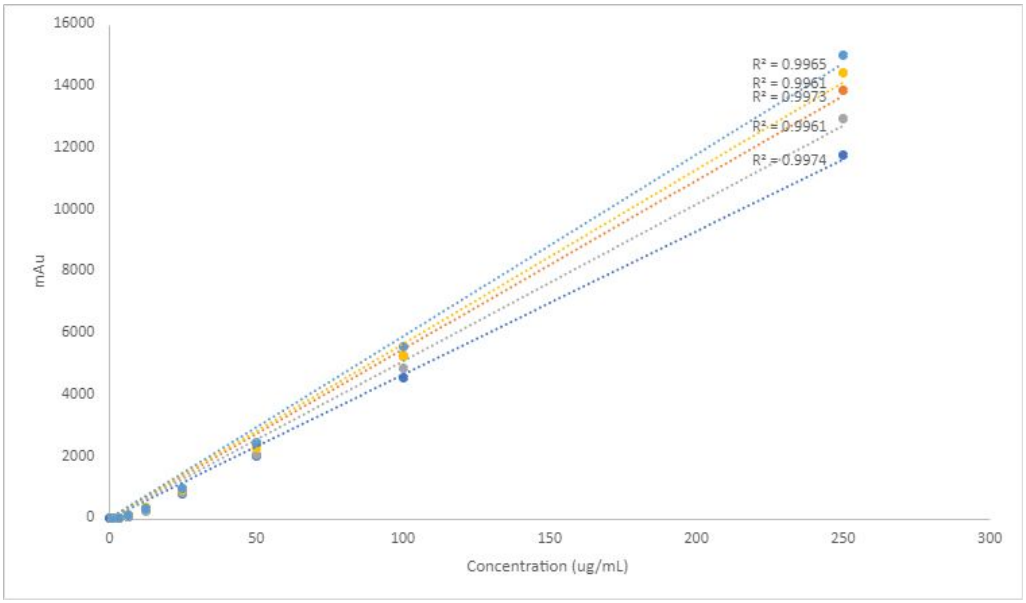


***Figure SI2:*** *Standard curves for 0, 1, 3.125. 6.25, 12.5, 25, 50, 100 and 250 µg/mL concentrations of recombinant human insulin diluted in PBS using HPLC detection, completed five times during the same HPLC run, used to determine intra-variability.*

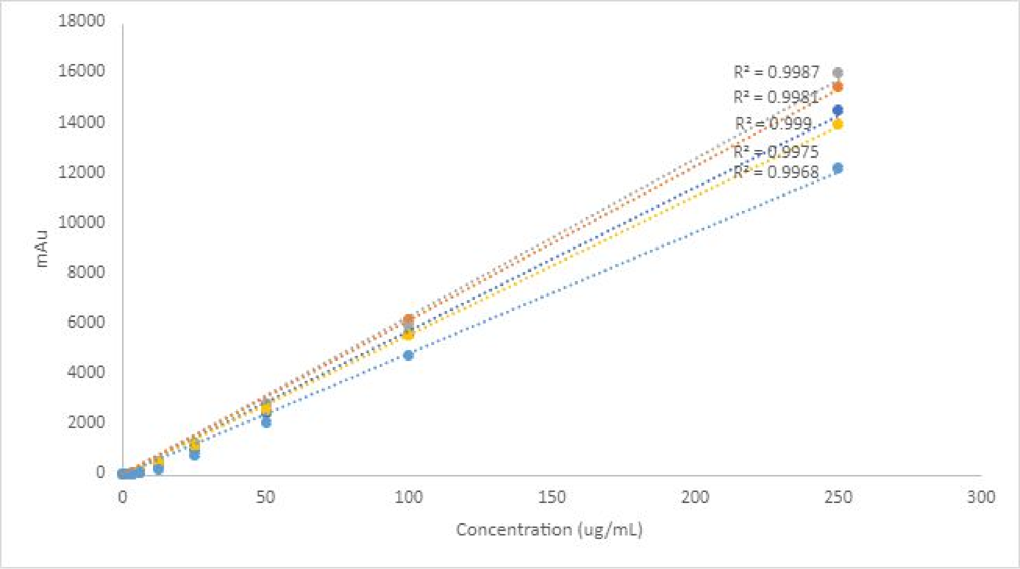


***Figure SI3:*** *Standard curves for 0, 1, 3.125. 6.25, 12.5, 25, 50, 100 and 250 µg/mL concentrations of recombinant human insulin diluted in PBS using HPLC detection, completed on five separate occasions, used to determine inter-variability.*

Using the signal-to-noise ratio, the limit of detection (LOD) and limit of quantification (LOQ) were determined. The LOD was determined as 1 µg/mL as the ratio frequently exceeded 3:1. An insulin concentration of 6.25 µg/mL reliably produced a STN ratio of over 10:1, whilst the lower concentration of 3.125 µg/mL did not. As such, 6.25 µg/mL was determined to be the LOQ, however it is likely that the true LOQ is lower than this.

HPLC of FITC-insulin

In terms of FITC conjugation, the reactivity of amines at different chain positions in insulin differs. The phenylalanine at position B1 is the most reactive, followed by the glycine at A1, then the lysine at B29. As such, referring to early work by Jacob et al. and Hentz et al., it has been shown before that the elution pattern remains the same during HPLC analysis (1,2). Always first to elute is any unconjugated insulin. This is followed by FITC-insulin singularly conjugated at B1, then FITC-insulin singularly conjugated at A1, then FITC-insulin conjugated at both B1 and A1 locations and, finally, FITC-insulin tri-

conjugated at all three locations, B1, A1 and B29. This facilitates the identification of how the FITC has conjugated with insulin for any FITC-insulin sample tested.

HPLC was employed to run a sample of the FITC-insulin at two different wavelengths, 215 nm and 495 nm respectively. 215 nm was used specifically to detect the insulin whilst 495 nm was to detect the FITC. Peaks were mapped according to the elution time. Several peaks had the same elution time at the different wavelengths, which suggested that the FITC reacted with the insulin at B1 (monosubstituted), A1 (monosubstituted) and at both the B1 and A1 (disubstituted) sites. A single peak observed on the 215 nm spectra suggested that a proportion of the insulin remained untagged by FITC. From the HPLC data, it appears that most of the unconjugated FITC had been successfully removed during the purification steps as a peak attributable to the FITC alone was not identified. Small peaks observed between the major peaks have been observed previously and are likely attributable to impurities in the FITC (1).

During the conjugation, a covalent thiourea bond is formed between the relevant amine group of the insulin and the isothiocyanate group of the FITC. Thiourea bonds are known to be stable. As such, it is unlikely that the thiourea bond would be broken upon injection into the skin, giving providing confidence that FITC-insulin is a valuable tool for understanding insulin distribution post administration.

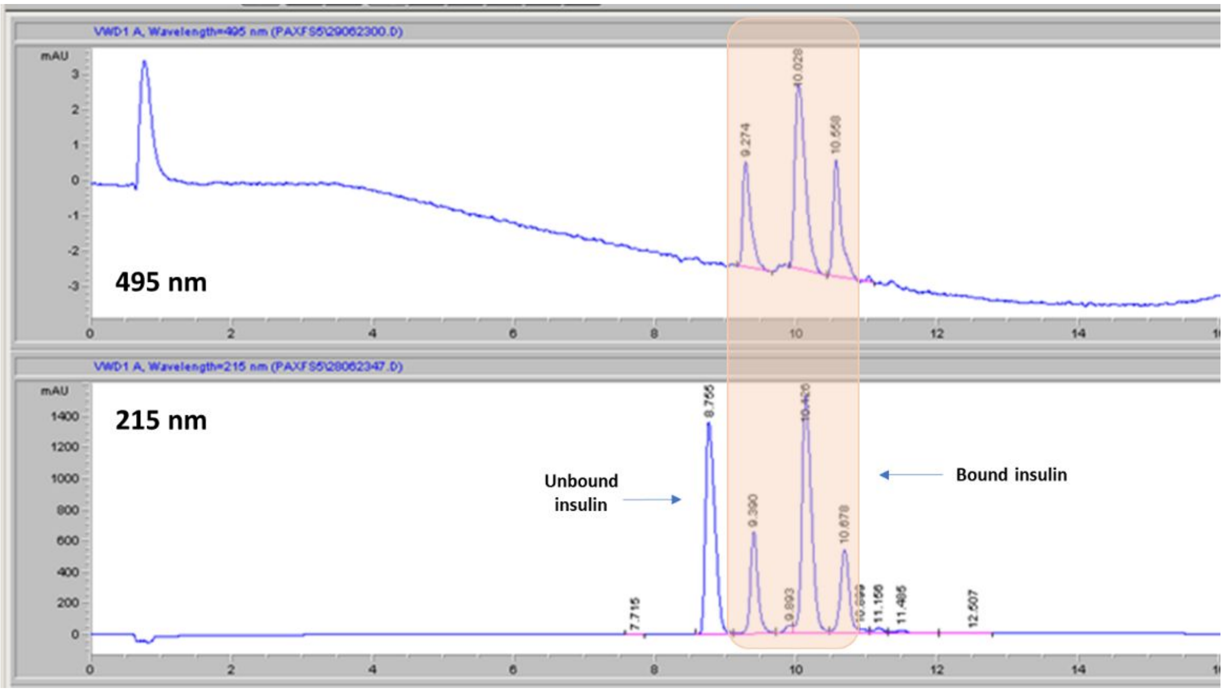


***Figure SI4:*** *HPLC chromatogram of FITC-insulin with UV detection at 215 nm (used to detect insulin) and 495 nm (used to detect FITC) demonstrating that the FITC has 3 different conjugates. Some insulin remains unbound to FITC as shown by the peak at 8.8 minutes.*

<u>Ex vivo insertion with commercially available hollow MNs</u>

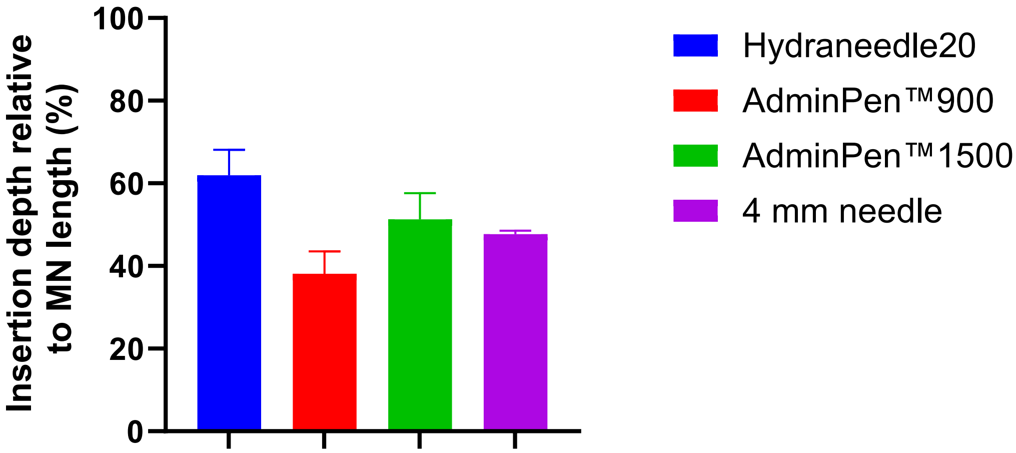


***Figure SI5:*** *An ex vivo study comparing the depth of insertion into porcine skin achieved relative to the length of the MN/needle inserted. The Hydraneedle20 (500 µm), AdminPen™900 (800 µm), AdminPen™1500 (1400 µm) and a 4 mm 31-gauge hypodermic needle were compared. The recorded insertion depth is considerably less*

*than the MN length in each case, demonstrating incomplete needle insertion into the skin. Data expressed as mean ± SEM, n=3.*

Full factorial design of experiments

MiniTab 21 Statistical Software was selected to design a full-factorial design of experiments (DOE). The aim was to understand whether there was a relationship between needle length and the hub diameter and how this affects the insertion profile. Five single hollow MNs, with dimensions listed in Table SI1, were produced based upon the upper and lower limits of parameters input into the software (needle height: lower limit 200 μm and upper limit 1000 μm, hub width: lower limit 2 mm, upper limit 10 mm), as described in Section 2.3. Each MN was inserted into *ex vivo* porcine skin 4 times and channel depth was measured using the methodology described in Section 2.5. A random run-order was dictated by the software to reduce bias, listed in Table SI3.

***Table SI1:*** *DOE run order generated in a random sequence by MiniTab 21 Statistical Software to reduce bias.*

| Run order | Needle length (μm) | Hub width (mm) |
|---|---|---|
| 1 | 200 | 10 |
| 2 | 1000 | 10 |
| 3 | 600 | 6 |
| 4 | 1000 | 2 |
| 5 | 600 | 6 |
| 6 | 200 | 2 |
| 7 | 1000 | 2 |
| 8 | 600 | 6 |
| 9 | 1000 | 2 |
| 10 | 200 | 2 |
| 11 | 1000 | 2 |
| 12 | 600 | 6 |

| | | |
|---|---|---|
| 13 | 1000 | 10 |
| 14 | 1000 | 10 |
| 15 | 1000 | 10 |
| 16 | 200 | 2 |
| 17 | 200 | 10 |
| 18 | 200 | 2 |
| 19 | 200 | 10 |
| 20 | 200 | 10 |

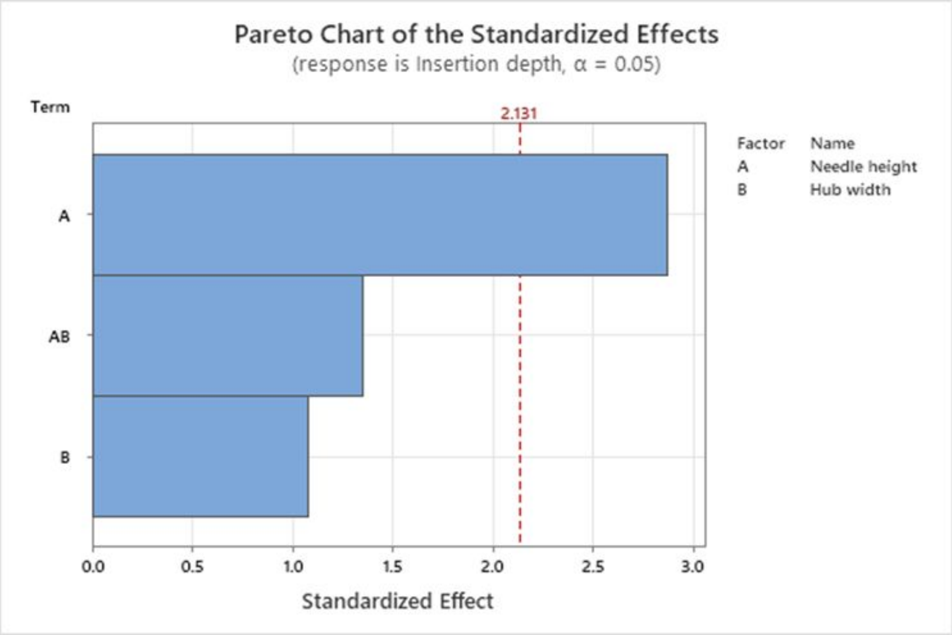


***Figure SI6:*** *Pareto chart of the standardised effects visualising whether hub width, needle height or the relationship between the two have a statistically significant effect on achievable insertion depth by crossing the red dashed line if so. Needle height was determined to have a statistically significant effect (p=0.012) whilst hub width and the interaction between hub width and needle height were not.*

<u>The effect of angle on insertion depth</u>

A mount was developed incorporating cork-covered wood, with the capability to vary the angle it rests. *Ex vivo* porcine skin was positioned on top of the cork before completing an insertion study (Section 2.5), allowing exploration of the effect of the angle of insertion on the insertion depth of a 1000 µm single hollow MN.

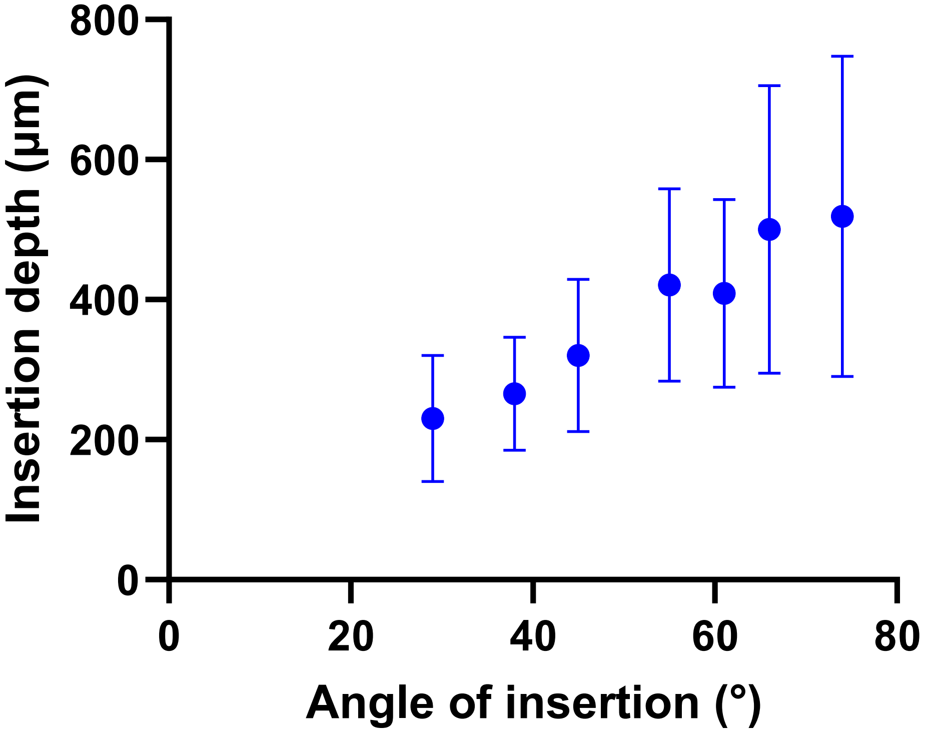


***Figure SI7:*** *Insertion of a single hollow MN into ex vivo porcine skin when the angle of insertion is varied. The relationship shows that insertion depth increased in relation to the angle. The considerable standard deviation of insertion depth achieved highlights that consistency of insertion depth remains an issue even as penetration depth increases (n=5 ± SD).*

References

1. Hentz NG, Richardson JM, Sportsman JR, Daijo J, Sittampalam GS. Synthesis and Characterization of Insulin-Fluorescein Derivatives for Bioanalytical Applications. Anal Chem [Internet]. 1997;69(24):4994–5000. Available from: https://pubs.acs.org/sharingguidelines

2. Jacob D, Joan Taylor M, Tomlins P, Sahota TS. Synthesis and Identification of FITC-Insulin Conjugates Produced Using Human Insulin and Insulin Analogues for Biomedical Applications. J Fluoresc. 2016 Mar 1;26(2):617–29.
